# High-Pressure X‑ray Diffraction Investigation of Fe_0.9_Al_0.1_VO_4_

**DOI:** 10.1021/acs.jpcc.5c01418

**Published:** 2025-04-17

**Authors:** Vinod Panchal, Pablo Botella, Neha Bura, Frederico G. Alabarse, Enrico Bandiello, Marco Bettinelli, Daniel Errandonea

**Affiliations:** † Department of Physics, Royal College, Mira Road, Thane, Mumbai 401107, India; ‡ Departamento de Física Aplicada, Instituto de Ciencias de Materiales, MALTA Consolider Team, Universitat de Valencia, Av. Dr. Moliner 50, 46100 Valencia, Spain; § 18474 Elettra Sincrotrone Trieste, Area Science Park T1-T2, 34149 Basovizza, Italy; ∥ Instituto de Diseño para la Fabricación y Producción Automatizada, MALTA Consolider Team, Universitat Politècnica de Valéncia, Cno. de Vera s/n, 46022 València, Spain; ⊥ Luminescent Materials Laboratory, Department of Biotechnology, University of Verona and INSTM, Strada Le Grazie 15, 37134 Verona, Italy

## Abstract

This study demonstrates that the influence of cationic composition on the phase behavior of vanadates under high pressure must be meticulously considered. In this investigation, we report an in situ high-pressure powder X-ray diffraction investigation on triclinic Fe_0.9_Al_0.1_VO_4_ (space group *P*1̅) up to 11 GPa. The structural sequence of Fe_0.9_Al_0.1_VO_4_ is different than that of FeVO_4_. Our analysis shows that Fe_0.9_Al_0.1_VO_4_ undergoes a first-order structural phase transition at 2.85 GPa to another triclinic structure described by the same space group with a volume collapse of ∼9%. At 6.1 GPa, we observed the onset of a second phase transition to a monoclinic structure (space group *P*2/*c*), with coexistence of both phases until 8.55 GPa. The transformation to the second phase is completed at 9.15 GPa, with a volume collapse of ∼13%. On release of pressure to ambient conditions, we have observed the coexistence of the second and first high-pressure phases. The compressibility of the three phases of the compound has been studied too. We have observed variations in structural sequence and compressibility behavior due to Al incorporation. Since electronic properties could be modified by tuning the crystal structure, the present results could have an impact on applications of the studied compound such as photocatalysis and batteries.

## Graphical abstract

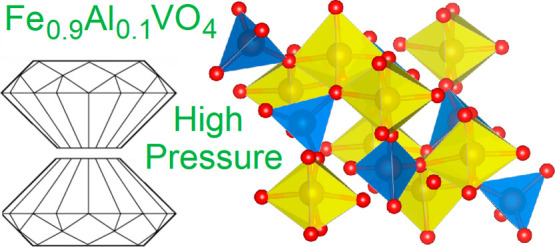

## 1 Introduction

Among the orthovanadates, FeVO_4_, due to its unique electronic, optical, and catalytic properties, finds many applications in various technologically important fields.^1,2^ Iron orthovanadate is considered a promising photocatalyst in environmental applications, particularly for wastewater treatment, due to its ability to efficiently degrade organic pollutants under visible light.^1,2^ FeVO_4_ is also a potential candidate for energy storage applications as a promising electrode material for supercapacitors,^3^ lithium-ion batteries, and sodium-ion batteries^4^ due to its excellent electrochemical properties, including high specific capacitance, good cycling stability, and redox activity, making it a potential candidate for high-power energy storage devices such as wearable electronics and hybrid electric vehicles. FeVO_4_ is also employed in gas-sensing applications, particularly for detecting gases such us ammonia (NH_3_), nitrogen dioxide (NO_2_), and carbon monoxide (CO), owing to its high sensitivity and selectivity.^5^ Due to its semiconducting properties and light absorption capabilities, it finds applications in solar cells and optoelectronic devices.^6^ FeVO_4_ doped with Cr shows an antiferromagnetic magnetization, making it relevant in spintronics and advanced electronic applications.^7^ It has also been observed that considerable enhancement in the spintronics,^7^ photocatalytic,^8,9^ and electrochemical^10^ properties of FeVO_4_ could be achieved by means of substitution of Fe with various trivalent transition and post-transition metals.^9^

At ambient conditions, FeVO_4_ crystallizes in a triclinic structure known as FeVO_4_-I (space group (SG) *P*1̅).^11,12^ The crystal structure is represented in Figure 1a. The unit cell has six formula units (*Z* = 6) and comprises 36 atoms, all of them occupying 2i Wyckoff positions, forming a complex network of chain-like motifs. In FeVO_4_, there are three distinct crystallographic sites for the Fe^3+^ ion. In two nonequivalent positions, it forms distorted FeO_6_ octahedra, whereas in the third position, it forms a distorted FeO_5_ trigonal bipyramid. These Fe–O polyhedral units form C-shaped chainlike networks running parallel to the crystallographic *c*-axis, with these chains interconnected by VO_4_ tetrahedra. Iron vanadate has multiple polymorphs, which differ in crystal structure and stability depending on temperature, pressure, and synthesis conditions. The well-known metastable polymorphs of FeVO_4_ include orthorhombic FeVO_4_-II (SG: *Cmcm*, 2 GPa −800 °C), orthorhombic FeVO_4_-III (SG: *Pbcn,* 6 GPa −750 °C), and monoclinic FeVO_4_-IV (SG: *P*2/*c*, 8 GPa −800 °C).^13^ These polymorphs have been experimentally recovered under ambient conditions. The monoclinic FeVO_4_-IV polymorph also has been reported to be synthesized at a much lower pressure of 5–5.5 GPa and 800 °C.^14^ In the past, numerous studies on triclinic FeVO_4_ have been carried out, which elucidated the structural phase transitions in this material under high-pressure conditions up to 12.3 GPa.^15–17^ It is worth mentioning a recent high-pressure single-crystal X-ray diffraction (XRD) and density functional theory (DFT) study in FeVO_4_ ^16^ which has presented pioneering findings with substantial evidence of structural phase transitions and provided valuable insights into the systematics of structural phase transitions in this material. According to this study, the FeVO_4_-I triclinic phase transforms at 2.1 GPa to another triclinic structure FeVO_4_-I′ (SG: *P*1̅) which was detected uniquely in this investigation. Additionally, a further transition to previously unknown monoclinic structure, FeVO_4_-II′ (SG: *C*2/*m*), was found at 4.8 GPa.^16^

**Figure 1:**
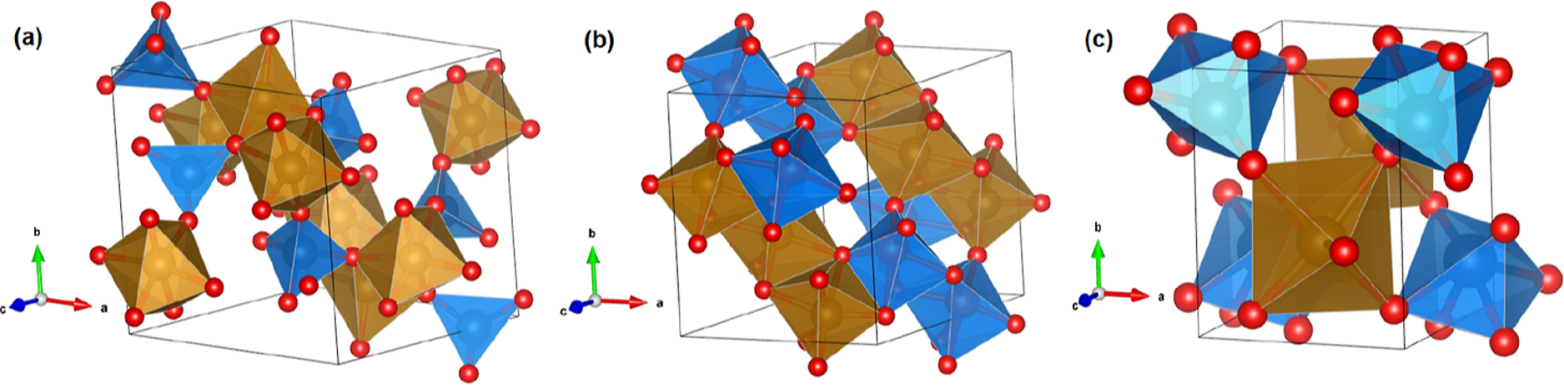
Crystal structure of phases I, I′, and IV of FeVO_4_ (Fe_0.9_Al_0.1_VO_4_). V (Fe/Al) coordination polyhedra are shown in blue (brown). Oxygen atoms are shown in red.

As discussed earlier, partially substituting Fe by a trivalent transition and post-transition elements enhances the properties of FeVO_4_. Till date, there is no high-pressure investigation reporting on partially substituted FeVO_4_ compounds. It is known that atomic substitution (chemical pressure) could affect the high-pressure behavior and properties of oxides. An investigation combining chemical and hydrostatic pressure contributes to deepen the understanding of the influence of pressure in vanadates, and it is useful for developing routes for synthesizing and tuning novel materials.^18–24^ Thus, in this investigation, we have carried out high-pressure synchrotron powder XRD measurements on Al-substituted FeVO_4_ up to 11 GPa to understand the nature of structural phase transitions in this material. Our findings could be valuable to tune the electronic, magnetic, and vibrational properties of this compound for practical applications.

## 2 Experimental Details

Single crystals of Fe_0.9_Al_0.1_VO_4_, up to 0.5 mm in size, were obtained by the flux method, using V_2_O_5_ as the self-flux. High-purity reagents Fe_2_O_3_, Al_2_O_3_, and V_2_O_5_ in powder form (Sigma-Aldrich) were mixed in a 0.9:0.1:2 molar ratio in a platinum crucible, which was sealed using a platinum cap. The closed crucible was heated in a furnace at 750 °C for 24 h, after which the furnace was cooled to 640 °C at 1.2 °C/h. Afterward, the crucible was removed from the furnace, allowed to cool at room temperature, and then uncapped. The crystals embedded in the flux were then separated from them by submerging and repeatedly flushing the whole crucible with hot nitric acid (1.5 M). Carefully selected crystals without evident flux inclusions were then used for our experiments. A finely ground powder was obtained from the crystals to perform high-pressure (HP) studies.

A membrane-type diamond-anvil cell (DAC), with diamond culets 400 μm in diameter, was used to generate the HP conditions. A stainless-steel gasket was first preindented to a 40 μm thickness, and after that, a 180 μm-diameter hole was drilled, in the center of the indentation, to serve as the sample chamber. The powdered sample was loaded together with a grain of copper (Cu) which was used to determine the pressure by means of the calibration provided by Dewaele et al.^25^ Pressure was determined with an accuracy better than 0.05 GPa. A 16:3:1 methanol–ethanol–water mixture was used as the pressure-transmitting medium (PTM). This PTM remains quasi-hydrostatic up to the highest pressure covered by this study.^26^ Room-temperature HP-XRD measurements were performed at the Xpress beamline of the Elettra synchrotron using a monochromatic wavelength of 0.4956 Å and a PILATUS 3S 6M detector. The powder XRD measurements were carried out in an angular dispersion configuration. The instrument was calibrated using cerium dioxide as a standard. The two-dimensional diffraction rings obtained from the detector were integrated using Dioptas^27^ to obtain the conventional intensity versus 2θ one-dimensional diffractograms. The structural analysis was performed by employing the Rietveld technique using GSAS.^28^ The background was adjusted using a Chebyshev polynomial function of the first kind, comprising eight coefficients, while the peak profiles were represented through a pseudo-Voigt function. In the refinements, we assumed that Al and Fe are distributed uniformly in all of the Fe positions of the structures of undoped FeVO_4_.

## 3 Result and Discussion

The XRD pattern of Al-substituted FeVO_4_ (Fe_0.9_Al_0.1_VO_4_) at 0.1 GPa recorded in the DAC together with Rietveld refinement is shown in Figure 2. The low-pressure phase can be assigned to a structure isomorphic to the FeVO_4_–I type-triclinic structure of pristine FeVO_4_ (space group *P*1̅, *Z* = 6). The unit-cell parameters at 0.1 GPa are found to be *a* = 6.698(5) Å, *b* = 8.020(7) Å, *c* = 9.328(5) Å, α = 96.55(1)°, β = 106.71(3)°, and γ = 101.48(3)°. The complete structural information, including atomic positions, is reported in Table 1. The goodness-of-fit parameters obtained were *R*_wp_ = 1.6% and *R*_p_ = 1.0%. The unit-cell parameters determined are slightly different than the values previously for the same structure in undoped FeVO_4_ ^11,12,15^ and by recent single-crystal measurements reported by Gonzalez-Platas et al. (CCDC 1987953).^16^ The unit-cell parameters determined in the previous study for FeVO_4_-I in the undoped sample are *a* = 6.7137(5) Å, *b* = 8.0609(5) Å, *c* = 9.3530(6) Å, α = 96.671(5)°, β = 106.645(6)°, and γ = 101.524(5)°. Thus, the substitution of 10% of Fe by Al induces a change in unit-cell parameters smaller than 0.5%, and a change smaller than 1% in the unit-cell volume.

**Figure 2:**
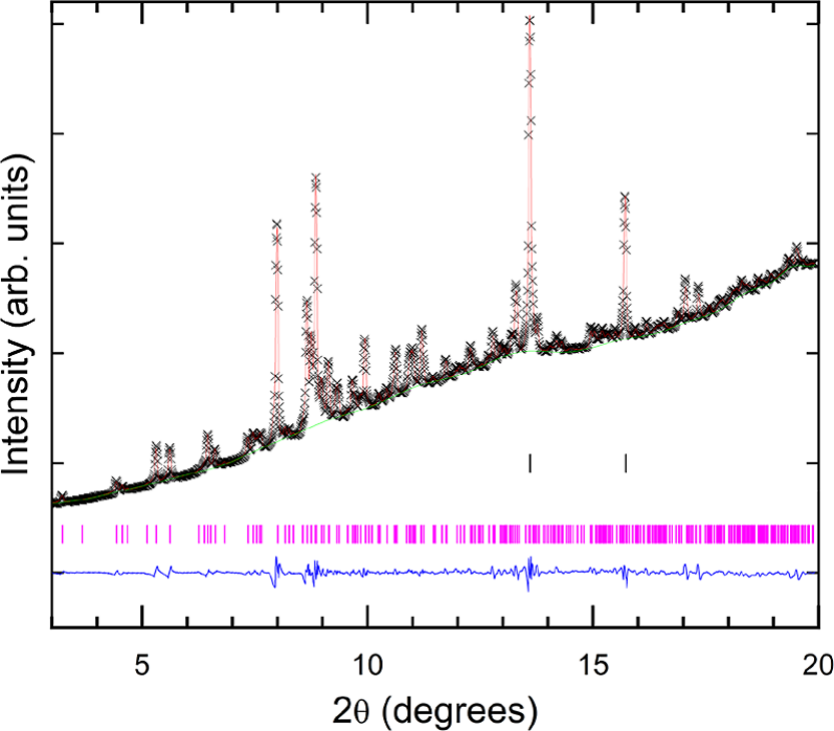
Rietveld refinement of the XRD pattern of triclinic Fe_0.9_Al_0.1_VO_4_ at ambient conditions (λ = 0.4956 Å). Data are shown as black crosses (×), while the red solid line represents the result of the refinement. The blue (green) line represents the residuals of the fitting (the background). Vertical pink (black) bars identify the Bragg reflections of Fe_0.9_Al_0.1_VO_4_ (copper).

**Table 1:** Crystal Structure of the Triclinic Phase I of Fe_0.9_Al_0.1_VO_4_ at 0.1 GPa and Room Temperature

*a* = 6.698(5) Å, *b* = 8.020(7) Å, *c* = 9.328(5) Å, α = 96.55(1)°, β = 106.71(3)°, and γ = 101.48(3)°, *V* = 462.4(4) Å^3^, *Z* = 6					
atom	site	*x*	*y*	*z*	*U*_iso_ (Å^2^)
Fe1/Al1	2i	0.7566(8)	0.6914(8)	0.9077(8)	0.0568
Fe2/Al2	2i	0.9793(8)	0.2996(8)	0.5144(8)	0.0756
Fe3/Al3	2i	0.4690(8)	0.8969(8)	0.7110(8)	0.0296
V1	2i	0.9973(8)	0.9970(8)	0.7560(8)	0.1040
V2	2i	0.2087(8)	0.6010(8)	0.8438(8)	0.0567
V3	2i	0.5266(8)	0.3036(8)	0.6351(8)	0.0551
O1	2i	0.064(3)	0.526(3)	0.673(3)	0.0594
O2	2i	0.771(3)	0.879(3)	0.762(3)	0.1712
O3	2i	0.132(3)	0.903(3)	0.675(3)	0.0347
O4	2i	0.460(3)	0.753(3)	0.890(3)	0.0207
O5	2i	0.624(3)	0.300(3)	0.459(3)	0.0983
O6	2i	0.933(3)	0.146(3)	0.665(3)	0.0184
O7	2i	0.257(3)	0.434(3)	0.926(3)	0.0245
O8	2i	0.176(3)	0.089(3)	0.932(3)	0.0900
O9	2i	0.072(3)	0.738(3)	0.964(3)	0.0168
O10	2i	0.247(3)	0.296(3)	0.545(3)	0.0792
O11	2i	0.634(3)	0.463(3)	0.749(3)	0.0235
O12	2i	0.535(3)	0.150(3)	0.749(3)	0.0542

Figure 3 shows the diffraction profiles at the selected pressures. There are no clearly visible changes in the diffraction patterns up to 2.35 GPa, and all the diffraction peaks could be indexed to the low-pressure triclinic phase, isomorphic to FeVO_4_-I. The diffraction peaks due to the pressure standard, i.e., copper, are marked as “Cu” in the diffraction profiles measured at 0.1, 6.1, and 0.1 MPa. The peaks from Cu are sharper than those from the sample and can be easily followed with pressure changes. A systematic shift was observed in all diffraction peaks due to lattice compression up to 2.35 GPa. However, at 2.85 GPa, we observed evident changes in the diffraction profile, indicative of the onset of a structural phase transition in Fe_0.9_Al_0.1_VO_4_.

**Figure 3:**
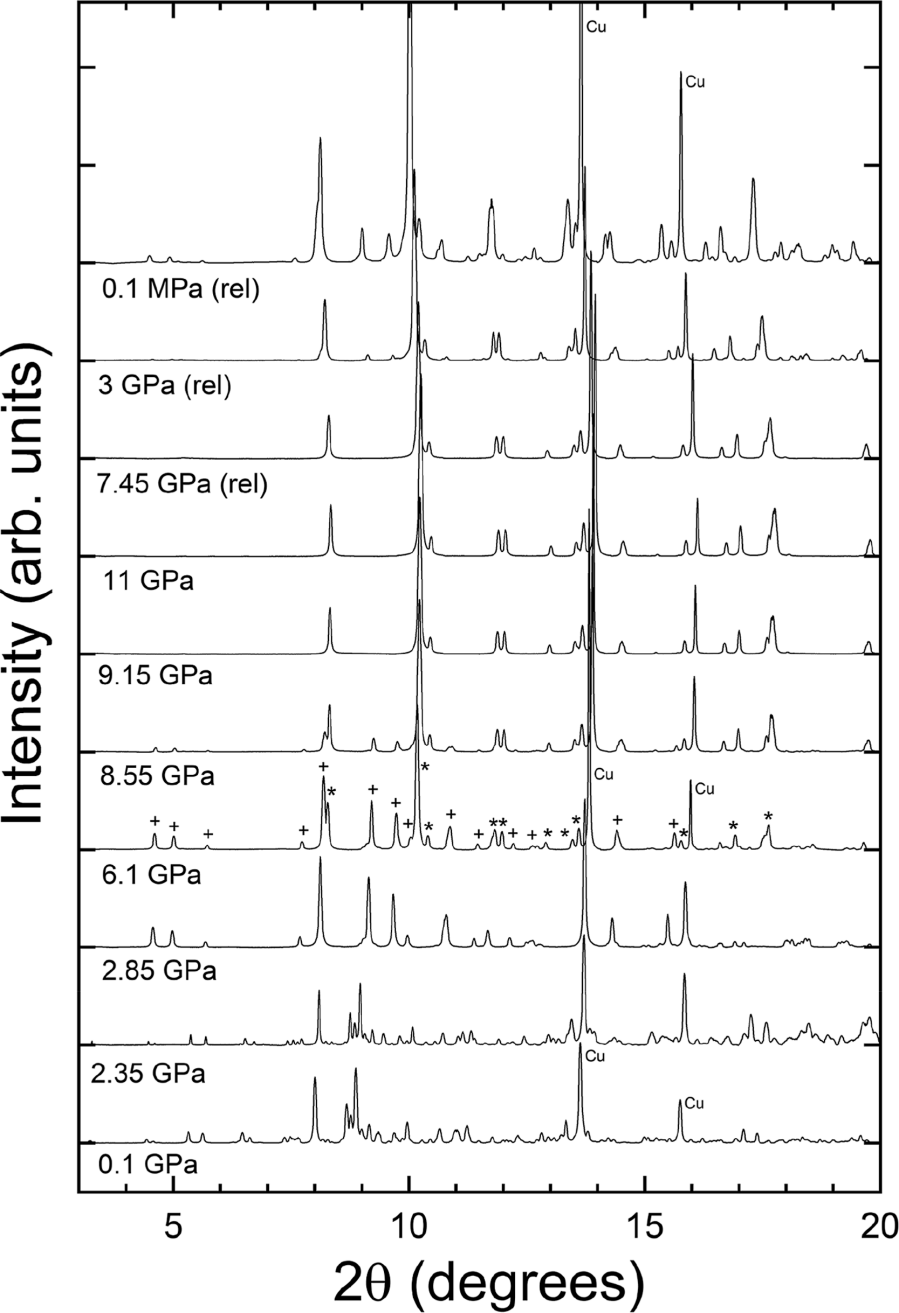
X-ray powder diffraction patterns of Fe_0.9_Al_0.1_VO_4_ at selected pressures (λ = 0.4956 Å). The patterns measured under pressure release are identified by (rel). Cu indicates the diffraction peaks of copper at 0.1 GPa, 6.1 GPa, and 0.1 MPa. At 6.1 GPa, (+) symbols identify diffraction peaks assigned to the triclinic HP phase, while (*) symbols identify emerging diffraction peaks assigned to the monoclinic HP phase.

As can be seen from Figure 3, the diffraction profile is completely transformed when the pressure is increased from 2.35 to 2.85 GPa with the appearance of several additional diffraction peaks. It is interesting to highlight that the replacement of Fe with Al (Fe_0.9_Al_0.1_VO_4_) leads to two opposite effects. On the one hand, it produces a decrease in the zero-pressure volume when compared with the pure FeVO_4_ crystal (1% decrease), equivalent to the effect of applying a hydrostatic pressure of 0.75 GPa. On the other hand, the transition pressures observed in the solid solution are observed at higher pressure than in FeVO_4_. The first is related to the chemical pressure induced by replacing Fe with Al. The second one is apparently contradictory. However, the increase in the pressure stability range due to the presence of impurities is not unexpected. Previous studies have shown that even minimal impurity concentrations, as low as 1 mol %, can enhance the stability range of various compounds.^29^ This seems to be related to the local disorder caused by impurities, which favor the resistance of materials to external stresses including pressure.^29^

The Rietveld refinement analysis presented in Figure 4 supports that the high-pressure phase has a triclinic structure isomorphic to FeVO_4_–I′ (space group *P*1̅, *Z* = 6), which in the following we will name simply as phase I’. The unit-cell parameters at 2.85 GPa are found to be *a* = 6.257(5) Å, *b* = 7.485(7) Å, *c* = 9.232(9) Å α = 98.87(7)°, β = 104.09(2)°, and γ = 95.60(7)°. This crystal structure is represented in Figure 1b. The complete structural information, including atomic positions, is tabulated in Table 2. The goodness-of-fit parameters obtained were *R*_wp_ = 2.0% and *R*_p_ = 1.2%.

**Figure 4:**
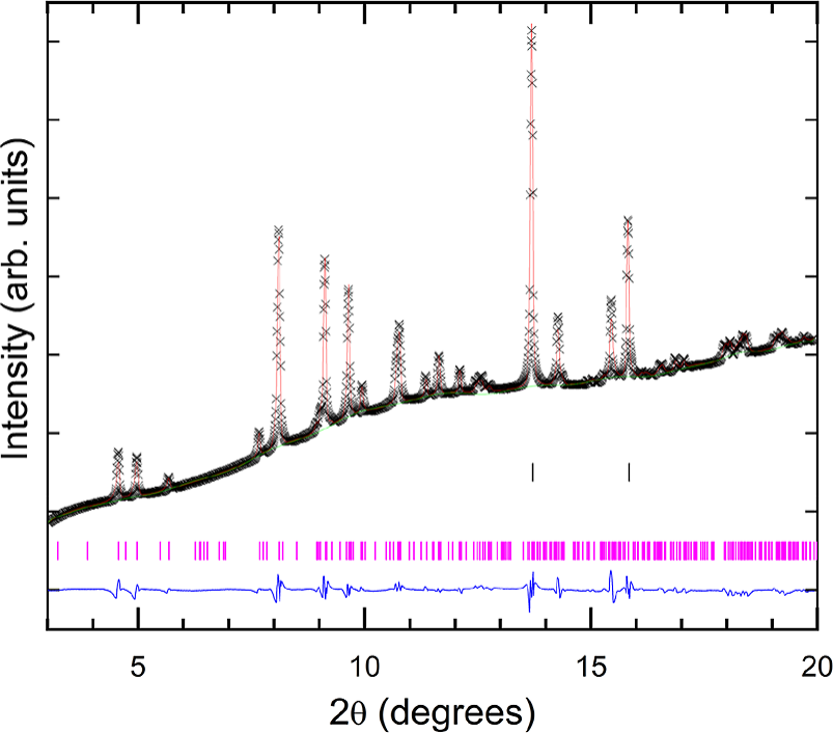
Rietveld refinement of the XRD pattern of the HP triclinic Fe_0.9_Al_0.1_VO_4_ (phase I′) at 2.85 GPa (λ = 0.4956 Å). Data are shown as black crosses (×), while the red solid line represents the result of the refinement. The blue (green) line represents the residuals of the fitting (the background). Vertical pink (black) bars identify the Bragg reflections of Fe_0.9_Al_0.1_VO_4_ (copper).

**Table 2:** Crystal Structure of the Triclinic Phase I′ of Fe_0.9_Al_0.1_VO_4_ at 2.85 GPa and Room Temperature

*a* = 6.257(5) Å, *b* = 7.485(7) Å, *c* = 9.232(9) Å α = 98.87(7)°, β = 104.09(2)°, and γ = 95.60(7)°. *V* = 410.2(4) Å^3^, *Z* = 6					
atom	site	*x*	*y*	*z*	*U*_iso_ (Å^2^)
Fe1/Al1	2i	0.262(1)	0.360(1)	0.662(1)	0.0221
Fe2/Al2	2i	0.568(1)	0.044(1)	0.755(1)	0.0332
Fe3/Al3	2i	0.903(1)	0.380(1)	0.092(1)	0.0544
V1	2i	0.935(1)	0.042(1)	0.323(1)	0.0231
V2	2i	0.596(1)	0.707(1)	0.986(1)	0.0196
V3	2i	0.765(1)	0.305(1)	0.558(1)	0.0162
O1	2i	0.899(5)	0.640(5)	0.912(5)	0.0750
O2	2i	0.576(5)	0.282(5)	0.642(5)	0.0235
O3	2i	0.161(5)	0.953(5)	0.305(5)	0.0052
O4	2i	0.939(5)	0.392(5)	0.694(5)	0.0094
O5	2i	0.401(5)	0.531(5)	0.865(5)	0.0688
O6	2i	0.704(5)	0.839(5)	0.198(5)	0.0434
O7	2i	0.379(5)	0.932(5)	0.623(5)	0.0029
O8	2i	0.697(5)	0.250(5)	0.956(5)	0.0157
O9	2i	0.714(5)	0.443(5)	0.462(5)	0.0177
O10	2i	0.927(5)	0.193(5)	0.210(5)	0.0284
O11	2i	0.046(5)	0.190(5)	0.520(5)	0.0201
O12	2i	0.605(5)	0.870(5)	0.886(5)	0.0385

The phase transition detected at 2.85 GPa is consistent with the earlier reported single-crystal XRD investigation in FeVO_4_.^16^ However, the transition pressure was reported as 2.1 GPa in FeVO_4_, which is 0.75 GPa lower as compared with our measurements. Thus, the partial substitution of Fe by Al extends the high-pressure stability of the low-pressure phase. It is worth noting that the observed structural phase transition involves changes in coordination of Fe/Al and V cations, and both the cations became octahedrally coordinated after the phase transition. This means that all the distorted FeO_5_/AlO_5_ trigonal bipyramids are converted to regular distorted octahedra. On the other hand, VO_4_ tetrahedra are also converted to distorted octahedra. The HP structure can be described by zigzag chains of edge-sharing FeO_6_ octahedra, which are connected via edge- and corner-sharing VO_6_ octahedra; see Figure 1c.

The change in coordination of Fe and V cations can affect the crystal field splitting, which in turn can modify the electronic energy levels of Fe and V. A higher coordination number can lower the crystal field stabilization energy, influencing bandgap tuning in semiconductor applications.^30,31^ Recent high-pressure studies on optical properties of FeVO_4_ ^17^ and InVO_4_ ^32^ attribute lowering of bandgap to change in coordination number due to alteration in the hybridization state of O 2p and V 3d and O 2p and Fe 3d orbitals, respectively. Given the fact that FeVO_4_ has a bandgap of 2.2 eV, it would not be surprising that compression could induce the metallization of Fe_0.9_Al_0.1_VO_4_. Our results suggest that it could be interesting to investigate in the future the effect of pressure on the band structure of Fe_0.9_Al_0.1_VO_4_.^33^

On further increase of pressure until 5.7 GPa, there are no evident changes in the diffraction profile other than the shift of peaks to higher angles due to lattice contraction. However, at 6.1 GPa, noticeable changes in the diffraction profile suggest the onset of a second phase transition. The extra peaks that indicate the existence of a second high-pressure phase are marked with the symbol “*” in Figure 3, while those originated by phase I′ are marked with the symbol “+”. Both phases coexist up to 8.55 GPa, and the transformation completes only at 9.15 GPa. This indicates that substitution of 10% Fe by Al has extended the range of stability of phase I′ from 4.8 to 8.55 GPa.

The partial substitution of Fe by Al affects not only the stability of phase I′ but also the structural sequence. Instead of the I′–II′ transition found in FeVO_4_,^16^ in Fe_0.9_Al_0.1_VO_4_, we found a different second HP phase. The Rietveld refinement presented in Figure 5 supports that the second high-pressure phase could be assigned to a monoclinic structure isomorphic to FeVO_4_-IV (space group *P*2/*c*, *Z* = 2), which we will call phase IV in the following, for consistence with the notation of previous studies in FeVO_4_.^15,16^ The unit-cell parameters determined at 9.15 GPa are found to be *a* = 4.385(1) Å, *b* = 5.452(3) Å, *c* = 4.796(7) Å, and β = 90.2(1)°. This HP structure resembles the wolframite phase previously found in CrVO_4_.^34^ The complete structural information, including atomic positions, is reported in Table 3. The goodness-of-fit parameters obtained were *R*_wp_ = 2.33% and *R*_p_ = 0.94%.

**Figure 5:**
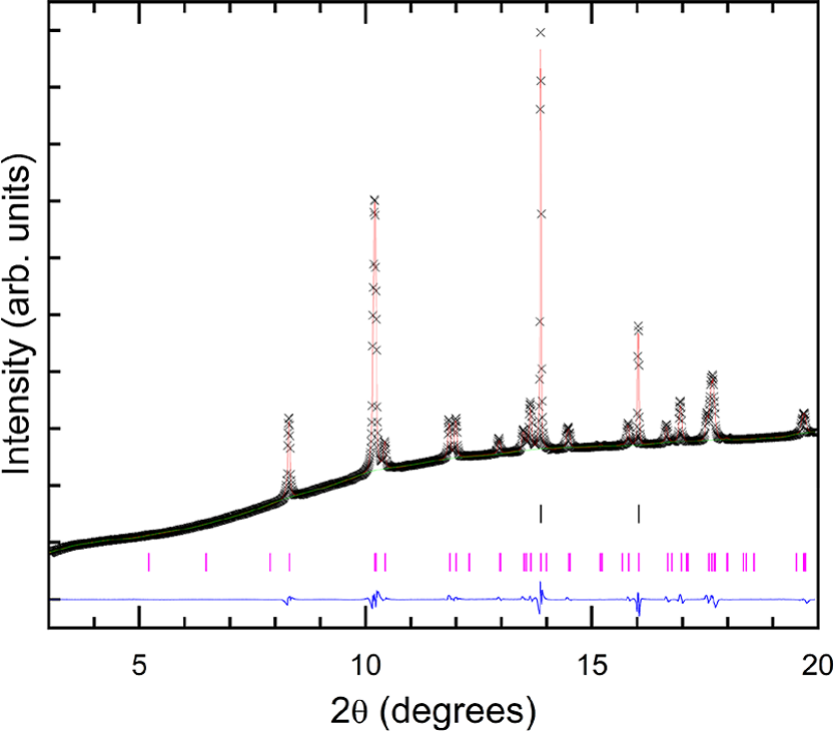
Rietveld refinement of the XRD pattern of the monoclinic phase IV structure at 9.15 GPa (λ = 0.4956 Å). Data are shown as black crosses (×), while the red solid line represents the result of the refinement. The blue (green) line represents the residuals of the fitting (the background). Vertical pink (black) bars identify the Bragg reflections of Fe_0.9_Al_0.1_VO_4_ (copper).

**Table 3:** Crystal Structure of the Monoclinic Phase IV of Fe_0.9_Al_0.1_VO_4_ at 9.15 GPa and Room Temperature

*a* = 4.385(1) Å, *b* = 5.452(3) Å, *c* = 4.796(7) Å, and β = 90.2(1)°. *V* = 114.6(2) Å^3^, *Z* = 2					
atom	site	*x*	*y*	*z*	*U*_iso_ (Å^2^)
Fe/Al	2f	0.5000	0.670(2)	0.2500	0.0104
V	2e	0.0000	0.185(2)	0.2500	0.0149
O1	4g	0.217(9)	0.105(9)	0.941(9)	0.0139
O2	4g	0.248(9)	0.368(9)	0.408(9)	0.0161

An earlier single-crystal XRD and DFT calculation investigation on FeVO_4_ has reported a similar transition at 4.8 GPa from FeVO_4_-I′ to FeVO_4_-II′^16^ before the transformation to FeVO_4_-IV. Thus, in this work, we have demonstrated that substituting Fe by Al in FeVO_4_ Al considerably increases the pressure range of stability of phase I′ and alters the structural phase sequence which is I–I′–IV in Fe_0.9_Al_0.1_VO_4_ instead of I–I′–II′–IV as in FeVO_4_. In the second structural transition, I′–IV, there is no change in the coordination of the cations. The Fe/Al cation is octahedrally coordinated to six oxygen atoms, forming nearly regular octahedra. The FeO_6_ and VO_6_ octahedra share edges with octahedral units of the same cations and are connected via edge-sharing with the octahedra of the other cation forming alternating zigzag chains.^34^ The HP monoclinic phase IV remained stable until 11 GPa, which is the highest pressure of these measurements. On release of the pressure, the structure reverts to the HP triclinic phase I′ at 3 GPa. On further release of pressure to ambient conditions, both phases I′ and IV coexisted, suggesting that phase transition is not reversible. The observed nonreversibility of the transitions, along with the discontinuity in the unit-cell volume identified at each transition (as will be demonstrated below), suggests that both transitions exhibit first-order characteristics.

Figure 6a–d shows the pressure dependence of the lattice parameters of Fe_0.9_Al_0.1_VO_4_ until 11 GPa. Since the crystal structures of the three phases of this compound are triclinic and monoclinic, to analyze their compressibility, we have used the eigenvalues and eigenvectors (principal axes) of the compressibility tensor^35^ obtained using the only tool PASCal.^36^ For this analysis, we used results from the compression and decompression cycles. The results are given in Table 4, where e_λi_ represents the direction of the principal axes of compressibility and κ_λi_ is the magnitude of the corresponding linear compressibility.

**Figure 6:**
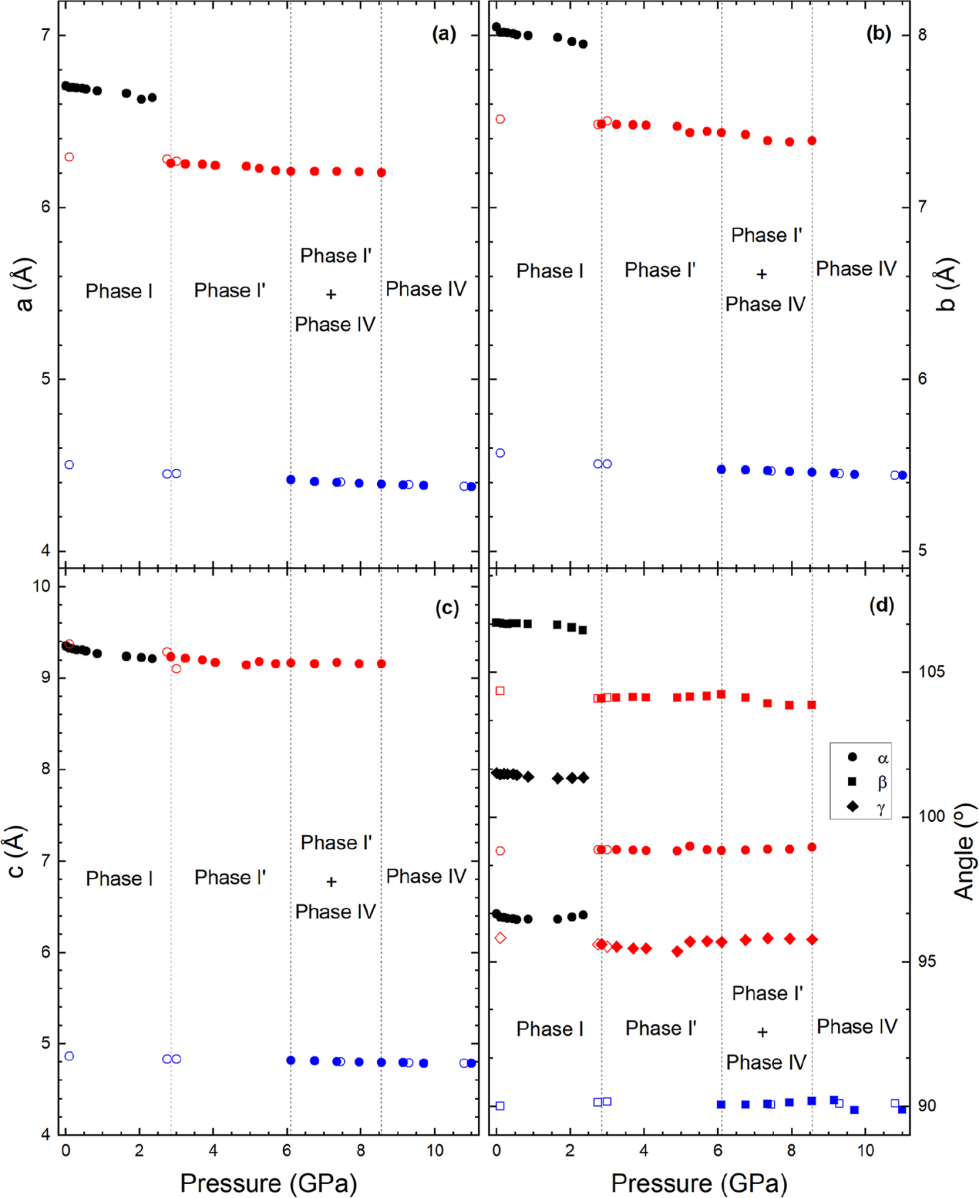
Pressure dependence of unit-cell parameters of Fe_0.9_Al_0.1_VO_4_. Black, red, and blue circles are used for phases I, I′, and IV. Solid symbols are from compression experiments and empty symbols from decompression experiments. The symbols used for different angles are identified in the legend.

**Table 4:** Principal Axes of Compressibility and the Corresponding Compressibility for the Three Polymorphs of Fe_0.9_Al_0.1_VO_4_

phase	phase I	phase I′	phase IV
e_λ1_	(16̅8)	(0110)	(100)
κ_λ1_ (in GPa^–1^)	6.6(8) 10^–3^	1.2(6) 10^–3^	1.8(1) 10^–3^
e_λ2_	(193̅)	(85̅1)	(010)
κ_λ2_ (in GPa^–1^)	3.6(1) 10^–3^	2.1(2) 10^–3^	1.4(1) 10^–3^
e_λ3_	(332)	(772)	(001)
κ_λ3_ (in GPa^–1^)	0.5(4) 10^–3^	2.1(5) 10^–3^	1.2(1) 10^–3^

We found that in phase I, Fe_0.9_Al_0.1_VO_4_ compression is highly anisotropic. The most compressible direction is (16̅8) with a compressibility 1 order of magnitude larger than the less compressible axis (332), which surprisingly has a linear compressibility as low as that of diamond. The isomorphic structure FeVO_4_-I has been also found to have a highly anisotropic compressibility;^16^ however, the principal axes of compressibility differ from those we determined for phase I in Fe_0.9_Al_0.1_VO_4_. In the HP triclinic phase of Fe_0.9_Al_0.1_VO_4_ (phase I′), the anisotropy of compressibility is reduced with two axes with a compressibility of 2.1 × 10^–3^ GPa^–1^ and the less compressible axis with a compressibility of 1.2 × 10^–3^ GPa^–1^. In the HP monoclinic phase of Fe_0.9_Al_0.1_VO_4_, the anisotropy is even more reduced. It is noticeable that in this phase, the β angle is always within 90.2 and 90° being the structure of compounds isomorphic to phase IV considered as pseudo-orthorhombic in the literature.^37^ A consequence of the small deviation of the β angle from 90° is the fact that the principal axes of compressibility are aligned with the crystallographic axes.

Figure 7 shows the *P*–*V* data for the three polymorphs of Fe_0.9_Al_0.1_VO_4_. In the figure, the compressibility of the studied compound decreases in the successive phase transitions. This observation is consistent with the successive collapses of the volume (9% and 13%, respectively). The abrupt changes in the volume and the irreversibility of the transitions indicate that the two transitions are first order in nature. The *P*–*V* data were fitted to the second-order Birch–Murnaghan equation of state (*K*_0_^′^ = 4)^38^ using EosFit7-GUI^39^ and considering both the bulk modulus (*K*_o_) and volume (*V*_o_) at 0 GPa as free parameters. The results of the fitting are summarized in Table 5. For the triclinic phase I of Fe_0.9_Al_0.1_VO_4_, a bulk modulus of 67(4) GPa is found, which is at least 12% smaller compared to the bulk modulus of FeVO_4_-I (see Table 5).^15,16^ For the first high-pressure phase, the EOS fit gives a bulk modulus of 132(11) GPa, which is at least 21% smaller than the bulk modulus of FeVO_4_-I′ (see Table 5).^16^ Finally, for the second high-pressure phase, the EOS fit gives a bulk modulus of 150(9) GPa, which is at least 14% smaller than the bulk modulus of FeVO_4_-IV. Thus, substituting Fe by Al has altered not only the structural sequence of the compound but also its elastic properties. This observation is consistent with the fact that Al–O bonds are more compressible than Fe–O,^40^ as shown by the fact that Al_2_O_3_ has a bulk modulus of 250 GPa and γ-Fe_2_O_3_ a bulk modulus of 303 GPa.

**Figure 7:**
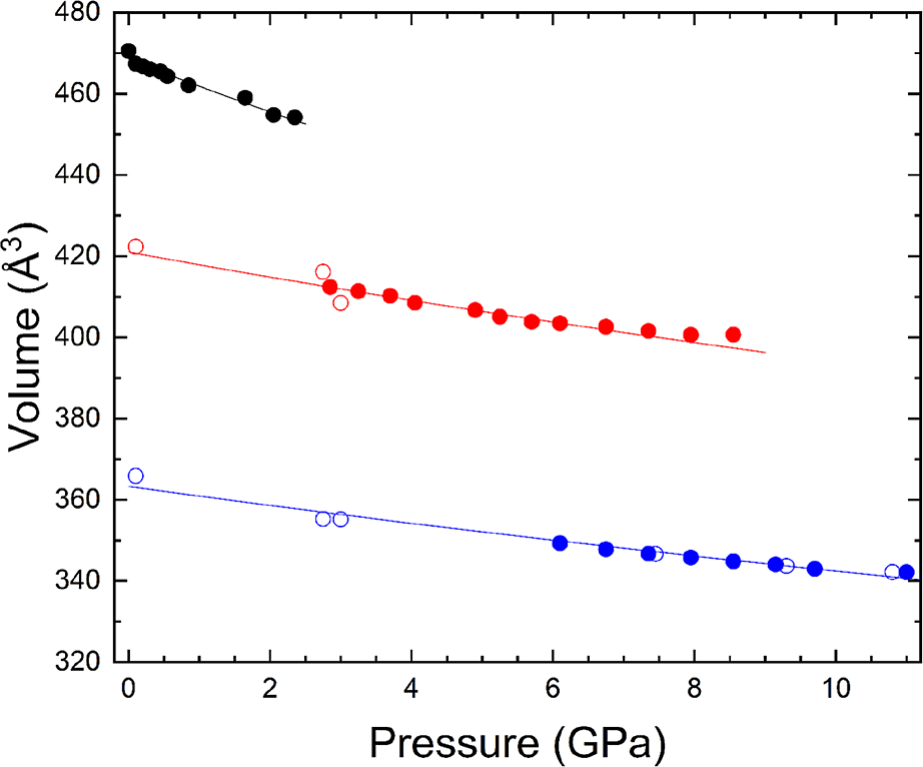
Volume versus pressure results for Fe_0.9_Al_0.1_VO_4_. Black, red, and blue circles are used for phases I, I′, and IV, respectively. Solid symbols are from compression experiments and empty symbols from decompression experiments. Solid lines represent Birch–Murnaghan fits to the data. The unit-cell volume of phase IV was multiplied by three to facilitate the comparison.

**Table 5:** Bulk Modulus (*K*_o_) and Volume (*V*_o_) at 0 GPa for the Three Polymorphs of Fe_0.9_Al_0.1_VO_4_ and the Same Polymorphs of FeVO_4_

phase	Fe_0.9_Al_0.1_VO_4_		FeVO_4_	
	*V*_*o*_ present study (Å^3^)	*K*_o_ present study (GPa)	*K*_o_ previous studies (GPa)	reference
phase I	468.6(6)	67(4)	80(4)	single-crystal XRD^16^
			87.3	DFT calculations^16^
			76(3)	powder XRD^15^
phase I′	421(2)	132(11)	181(17)	single-crystal XRD^16^
			167.5	DFT calculations^16^
phase IV	363.3(9)	150(9)	174(8)	powder XRD^15^
			206.7	DFT calculations^15^

## 4 Conclusions

Our high-pressure synchrotron powder XRD experiments on Al-substituted triclinic FeVO_4_ (Fe_0.9_Al_0.1_VO_4_) suggest that at room temperature, the low-pressure triclinic phase undergoes a first-order phase transition at 2.85 GPa to another triclinic structure isomorphic to FeVO_4_-I′ with ∼9% volume collapse and a change in coordination of Fe and V cations. The onset of a second phase transition is observed at 6.1 GPa with coexistence of both phases until 8.55 GPa. The second high-pressure phase is identified as a monoclinic structure isomorphic to FeVO_4_-IV. The transition completes at 9.15 GPa, and the second high-pressure phase remains stable up to 11 GPa. These transitions are found to be irreversible as the two high-pressure phases coexist at ambient pressure. The structural sequence induced by pressure deviates from the one found in FeVO_4_, as the transition pressures are different, and no intermediate phases are found between phase I′ and IV. In addition, we have observed an alteration of the compressibilities in all the polymorphs of Al-substituted FeVO_4_. In particular, the three polymorphs of Fe_0.9_Al_0.1_VO_4_ are more compressible than the isomorphous polymorphs of FeVO_4_. In summary, a 10% substitution of Fe with Al clearly affects the high-pressure behavior of FeVO_4_. The inclusion of crystal in the crystal structure affects the structural sequence, which is different in Fe_0.9_Al_0.1_VO_4_ than in FeVO_4_, and modifies the mechanical properties of FeVO_4_. The present study shows that the role of cationic composition in the phase behavior of vanadates at high pressures should be carefully considered when modeling them.

## Data Availability

The data that support the findings of this study are available from the corresponding author upon reasonable request.
