# Addition to “High Pressure X‑ray Diffraction Investigation of Fe_0.9_Al_0.1_VO_4_”

**DOI:** 10.1021/acs.jpcc.5c04957

**Published:** 2025-07-25

**Authors:** Vinod Panchal, Pablo Botella, Neha Bura, Frederico G. Alabarse, Enrico Bandiello, Marco Bettinelli, Daniel Errandonea

Due to an oversight the authors forgot to include a source of financial support in the acknowledgments. This oversight is corrected with this revision.

